# Supplementary material for: Implementing and Delivering Youth Mental Health Services: Approaches Taken by the Australian Primary Health Network ‘Lead Sites’
Source: Int J Environ Res Public Health. 2022 Aug 23;19(17):10494. doi: 10.3390/ijerph191710494 (PMC9517850; doi:10.3390/ijerph191710494)
Supplement: Supplementary file 1 [file ijerph-19-10494-s001.zip › ijerph-1839982-supplementary.pdf]

Supplementary Materials.

Focus group questions for Lead Site staff

1) Overall, which approaches, strategies or activities were most effective for the targeting and delivery of youth-severe services in your region? Any evidence that your youth-severe strategies are reaching the target group(s)?
   a) *Any examples of where clinical care is being effectively complemented by vocational, educational and parental support programmes?*
   b) *What effective linkages were formed with other regional youth-specific services, including those provided by states and territories, headspace, schools and other educational institutions?*

2) Overall, which approaches, strategies or activities were least effective for the targeting and delivery of services to young people with, or at risk of, severe mental illness?

3) What (new) factors have helped your PHN in procuring and delivering youth-severe services?

4) What (new) difficulties has your PHN experienced in procuring and delivering youth-severe services?

5) How has your PHN managed to overcome the main difficulties experienced in procuring and delivering youth-severe services?

6) What are the (early) impacts of youth-severe services?

7) How could planning, commissioning and implementing youth-severe services be improved in the future?
   a) *What factors are considered essential for effective youth-severe service delivery?*
   b) *Do you need different types of supports or resources from the Department?*

Focus group questions for external stakeholders

1) How were you or your organisation involved in PHNs commissioning services for youth with, or at risk of, severe mental illness?

2) What effects has the introduction of the new services for young people with, or at risk of, severe mental illness had on consumers and carers?
   a) Have you seen any evidence that services for young people with, or at risk of, severe mental illness have been effective? If so, please elaborate.

3) How has clinical care for young people with, or at risk of, severe mental illness been complemented by other programs important for young people, such as vocational, educational and parental support programs?

4) How might services for young people with, or at risk of, severe mental illness be improved in the future?
